# Impact of ambient air pollution on physical activity engagement among university students

**DOI:** 10.3389/fpubh.2024.1488115

**Published:** 2024-11-20

**Authors:** Kampanat Wangsan, Jinjuta Panumasvivat, Thiti Usanakul, Vorapat Sirivoravith, Supanut Rojanachai, Natchaphol Zheng, Chonlathee Boontan, Ratana Sapbamrer

**Affiliations:** ^1^Department of Community Medicine, Faculty of Medicine, Chiang Mai University, Chiang Mai, Thailand; ^2^Environmental and Occupational Medicine Excellence Center (EnOMEC), Department of Community Medicine, Faculty of Medicine, Chiang Mai University, Chiang Mai, Thailand; ^3^Faculty of Medicine, Chiang Mai University, Chiang Mai, Thailand; ^4^Melbourne School of Population and Global Health, University of Melbourne, Melbourne, VIC, Australia

**Keywords:** physical activity, PM2.5, air pollution, university students, motivation

## Abstract

**Introduction:**

PM2.5 poses significant health risks with prolonged exposure, potentially reducing physical activity levels. This study aims to investigate the impact of ambient PM2.5 levels on physical activity engagement among individuals.

**Methods:**

The retrospective cross-sectional study involved 423 students from Chiang Mai University residing there between January and August 2023. We used the validated Thai version of the International Physical Activity Questionnaire to assess physical activity intensity during high and low pollution periods. Individuals who engage in at least 150 min of moderate-intensity or 75 min of high-intensity physical activity per week meet the recommended physical activity guidelines. Multiple logistic regression analyzed air pollution's relation to physical activity intensity, and Cochran's *Q*-test compared activity levels across pollution periods.

**Results:**

Recommended physical activity prevalence was 76.36% during high PM2.5 and 71.63% during low PM2.5 periods, it showed higher physical activity during polluted periods (*p* = 0.049). Individuals' behavior showed a preference for indoor exercise (*p* < 0.001), consistent PM2.5 level checks (*p* < 0.001) during high PM2.5 periods. Internal motivation significantly associated with recommended physical activity in both low and high PM2.5 periods (aOR = 2.46, 95% CI = 1.14 – 5.27 and aOR = 4.00, 95% CI = 1.84 – 8.70, respectively). The outdoor exercise significantly associated with the recommended physical activity only during the low PM2.5 period (aOR = 1.72, 95% CI = 1.03 – 2.87).

**Conclusion:**

University students showed increased physical activity intensity during high PM2.5 periods, favoring indoor exercise in polluted environments. This behavioral shift highlights environmental pollution's influence on lifestyle choices and cornering on health outcomes. Government and university support is crucial for implementing measures to mitigate PM2.5 pollution, including promoting safe indoor exercise, enhancing pollution control measures, and developing air quality monitoring and warning systems.

## 1 Introduction

Fine Particulate matter (PM2.5) is one of the most significant ambient air pollution problems globally. Long-term exposure to air pollution heightens the likelihood of cardiovascular and respiratory illnesses, type 2 diabetes, various cancers, and premature death (1). According to the World Health Organization, the combined effects of ambient and household air pollution contribute to ~7 million premature deaths annually (2). Reducing aerosol levels to a safe threshold, indicated by an Air Quality Index (AQI) below 100, in developing nations could potentially save between 300,000 and 700,000 lives from premature mortality (3). Northern Thailand, especially Chiang Mai, faces significant air pollution issues, particularly with PM2.5. While WHO recommends PM2.5 levels not exceed 15 μg/m^3^ over 24 h, Chiang Mai frequently surpasses this by 7–10 times during January to April (4). Open burning of crop residues and forest fires, both locally and from neighboring countries, during the January to April are the main contributors to this problem (5, 6).

Similar to air pollution, physical inactivity is a significant factor contributing to and linked with premature deaths from non-communicable diseases (NCDs) (7). According to WHO Global Status Report on Physical Activity 2022, most people, including children and adults in both developed and developing countries, do not meet the recommended physical activity guidelines (8). These guidelines suggest engaging in at least 60 min of moderate to vigorous intensity physical activity daily, incorporating vigorous aerobic activities and strengthening exercises at least three times per week (9, 10). Regular physical activity offers numerous health advantages, such as lowering overall mortality rates and decreasing the risk of chronic illnesses like cardiovascular diseases, cancer, diabetes, and depression (11).

University students are at high risk for a sedentary lifestyle, spending most of their time in lecture rooms, seminar rooms, or at desks (12). They report lower physical activity levels compared to the general population (13, 14), which is linked to negative health outcomes such as lower quality of life, sleep disturbances, and decreased life satisfaction (13, 15, 16). The combined impact of air pollution and physical activity on cardiovascular disease (CVD) is emerging as a crucial public health concern among factors related to CVD (17, 18). While the advantages of physical activity are well-known, the balance between these benefits and the risks posed by exercising in polluted air conditions remains unclear. The health risks linked to air pollution are generally seen as increasing linearly with higher exposure levels, especially in low to moderate pollution scenarios, while the benefits of physical activity tend to increase in a curvilinear manner with greater engagement (19). This suggests that there may be a point where, with a certain level of background air pollution and physical activity, the risks could outweigh the benefits. The connection between air pollution and physical activity is complex and poorly understood. Public alerts regarding air quality, aimed at informing the public about harmful air pollution, could influence individuals' choices regarding physical activity (20).

With limited existing research on the relationships between PM2.5, physical activity, and sedentary behavior, the impact of air pollution on physical activity as a motivating or discouraging factor for individuals in affected areas is not well-understood. The aim of this study is to investigate whether ambient PM2.5 level influences individuals' choices to participate in outdoor physical activities.

## 2 Materials and methods

### 2.1 Study design and population

**Figure 1:**
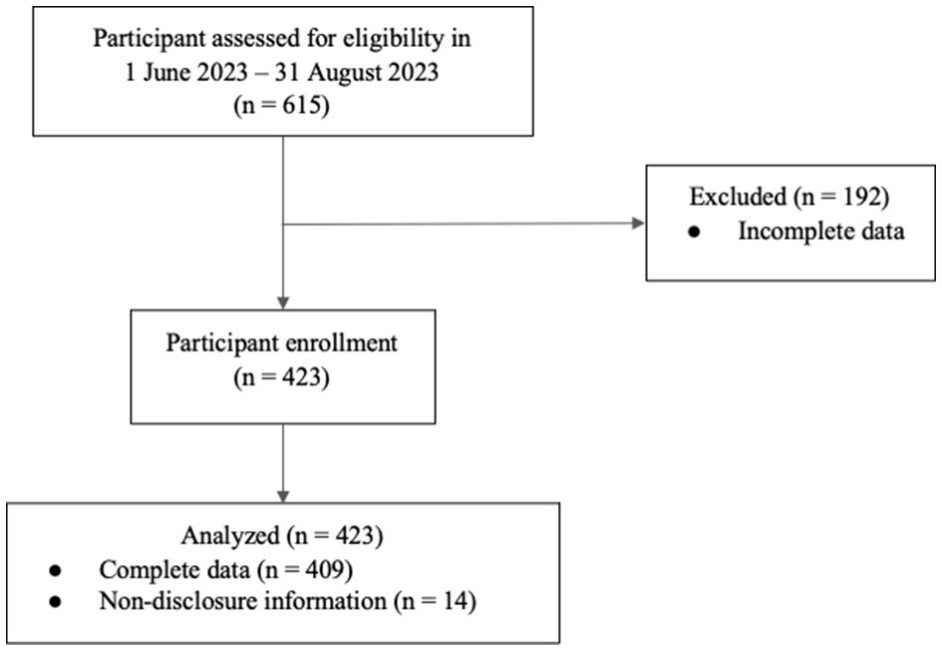
Flow chart illustrating the amount screening participants based on inclusion-exclusion criteria.

The study was designed as a retrospective cross-sectional study conducted from June to August 2023 during the low PM2.5 period, with data collected through a one-time questionnaire. Participants were asked about their physical activity during both the high and low PM2.5 period. Using a convenience sampling method, volunteers participated voluntarily by accessing the QR code or the provided link to an online survey. The study was promoted via social media platforms and was also announced to each faculty at Chiang Mai University. The inclusion criteria were Chiang Mai University students who agreed to participate in the study and had resided in Chiang Mai between January and August 2023. Participants who did not complete all the questions were excluded from the study. The methods for screening qualified participants are illustrated in Figure 1 below. Non-disclosure information refers to participants choosing not to provide details which is exercise location and PM2.5 level checks.

### 2.2 Data collection

To assess high and low PM2.5 periods, hourly PM2.5 concentrations in Chiang Mai city were collected via the Pollution Control Department's online reports from January to August (21). The PM2.5 level was categorized into two phases: January to April, marked as the high PM2.5 period, and May to August, marked as the low PM2.5 period. This classification was based on the Pollution Control Department's PM2.5 decadal trend in Chiang Mai and the report by Sapbamrer et al. (5, 21). The individual data consist of six parts: (1) Demographic data, (2) Health information, (3) Intensity of physical activity in the high pollution period, (4) Intensity of physical activity in the low pollution period, (5) Physical activity motivation, and (6) their perspectives on the correlation between air pollution and individual health including location of exercise and PM2.5 concentration check during high and low PM2.5 period. The overall questionnaire was validated by two community medicine experts.

Motivations for physical activity were categorized as internal or external and assessed with Yes/No responses. Internal motivations, which refers to engaging in an activity because it is inherently satisfying or self-motivated, included factors such as improving immunity and health, enhancing physical appearance, weight loss, mental health, better sleep, increasing strength, and rehabilitation. External motivations, which refers to engaging in an activity to external stimuli, included social interactions, invitations from others, travel, team participation, advice from specialists, and inspiration from celebrities or environments. Individuals reporting “yes” to any motivation were considered motivated.

### 2.3 Physical activity level

The intensity of physical activity was evaluated using the Thai version of the International Physical Activity Questionnaire (IPAQ), developed by Rattanawiwatpong et al. (22). The questionnaire's validity is indicated by values of Spearman's correlation coefficients (rs = 0.32), weighted kappa (k = 0.22), proportion of agreement (*p* = 0.65), and reliability is indicated by Intraclass Correlation Coefficients (ICC = 0.69). The parameters indicate that the Thai version of IPAQ (22) demonstrates acceptable validity and reliability for assessing physical activity. The questionnaire comprises seven questions, including: total time spent on high-intensity physical activity, moderate physical activity, and walking, each measured in days per week and minutes per day, and total time spent on sitting on a weekday.

All data from the IPAQ will be analyzed using the IPAQ scoring protocol (22, 23) which employs a continuous variable score called metabolic equivalent of task (MET). Total MET minute per week were calculated as follows: (minutes per day of walking × days per week with walking × 3.3) + (minutes per day of moderate-intensity activity × days per week with moderate-intensity activity × 4.0) + (minutes per day of vigorous activity × days per week with vigorous activity × 8.0).

Physical activity intensity is categorized into three groups based on the following criteria (23): (1) High levels of physical activity: Engaging in high-intensity activity at least 3 days a week with a total score of at least 1,500 MET min per week, or a combination of activities totaling at least 3,000 min per week. (2) Moderate levels of physical activity: Meeting one of four criteria, including engaging in high-intensity activity on at least 3 days a week, participating in moderate-intensity activity on at least 5 days a week, walking for at least 30 min per day, or achieving at least 600 min per week through any combination of these activities. (3) Low levels of physical activity are attributed to individuals who do not meet any of the aforementioned criteria.

For multiple logistic regression, physical activity was divided into a binary outcome. Individuals with moderate to high physical activity scores were classified as meeting the recommended physical activity per WHO guidelines (24), while those with low physical activity were categorized as having poor physical activity, serving as the reference group.

### 2.4 Statistical analysis

The descriptive statistical model, the continuous data were analyzed using the central tendency model, while categorized data were assessed using percentages. To compare the intensity of physical activity between two different periods—high and low PM2.5 periods—Cochran's *Q*-test was used. Multiple logistic regression was utilized to examine the association between recommended physical activity levels and potential factors from the extensive review literature such as age, sex, BMI, underlying disease, smoking, alcohol use, mental health, sitting time, faculty, income, internal/external motivation, PM2.5 level checks, and exercise location. The outcome variable, recommended physical activity, was analyzed separately for high and low PM2.5 periods. For 14 non-disclosure information, we analyzed only the available data. The significance level was set at a *p*-value of < 0.05 and Confidence Interval > 95%. STATA version 16.0 (StataCorp LLC, College Station, Texas, USA) was assessed for evaluating all the data.

This study and the protocol was approved by the Research Ethics Committee, Faculty of Medicine, Chiang Mai University, Thailand (Study code: COM-2566-09474). Informed consent was obtained from all individual participants included in the study. All procedures were performed in compliance with the World Medical Association Declaration of Helsinki.

## 3 Results

### 3.1 Participant's characteristics

A total of 423 participants, with an average age of 20.0 years, comprised the study group, among which 58.6% were female and 41.4% were male. The majority of participants were non-smokers without underlying diseases, enrolled in the Health Sciences faculty. Approximately half of the participants reported consuming alcohol, while the remaining half did not. The mean BMI among participants was 21.7 (4.2) kg/m^2^.

Regarding physical activity, the majority reported a moderate level (52.0%), followed by low and high (19.6%) levels. The number of hours spent in sitting per day varied across the different physical activity levels. Sitting time varied across these levels, with those reporting moderate physical activity engaging in 8.1 h per day, followed by high level (7.7 h) and low level (7.5 h). Additional demographic characteristics and health parameters are presented in Table 1.

**Table 1:** Factors associated with the intensity of physical activity among the participants in low PM2.5 period.

**Variable**		**Total**	**Low level of PA**	**Moderate level of PA**	**High level of PA**	***p*-value**
		***N* = 423**	***N* = 120**	***N* = 220**	***N* = 83**	
		***N*** **(%)**	***N*** **(%)**	***N*** **(%)**	***N*** **(%)**	
Age (years) (Mean ± SD)		20.0 (1.9)	19.8 (1.8)	20.1 (2.0)	20.2 (1.7)	0.27
Sex	Male	175 (41.4%)	45 (37.5%)	85 (38.6%)	45 (54.2%)	**0.030** ^*^
	Female	248 (58.6%)	75 (62.5%)	135 (61.4%)	38 (45.8%)	
BMI (kg/m^2^) (Mean ± SD)		21.7 (4.2)	21.6 (4.3)	21.7 (4.4)	21.6 (3.3)	0.99
Faculty	Non-health sciences	98 (23.2%)	33 (27.5%)	52 (23.6%)	13 (15.7%)	0.14
	Health sciences	325 (76.8%)	87 (72.5%)	168 (76.4%)	70 (84.3%)	
Income (Baht/month)	< 5,000 Baht	59 (13.9%)	17 (14.2%)	29 (13.2%)	13 (15.7%)	0.40
	5,000–10,000 Baht	250 (59.1%)	78 (65.0%)	124 (56.4%)	48 (57.8%)	
	>10,000 Baht	114 (27.0%)	25 (20.8%)	67 (30.5%)	22 (26.5%)	
Sufficient income	Sufficient	356 (84.2%)	96 (80.0%)	186 (84.5%)	74 (89.2%)	0.22
	Insufficient	67 (15.8%)	24 (20.0%)	34 (15.5%)	9 (10.8%)	
Underlying disease	Have U/D	69 (16.3%)	19 (15.8%)	34 (15.5%)	16 (19.3%)	0.68
	No U/D	354 (83.7%)	101 (84.2%)	186 (84.5%)	67 (80.7%)	
Smoking status	Smoking	2 (0.5%)	1 (0.8%)	1 (0.5%)	0 (0.0%)	0.57
	Non-smoking	413 (97.6%)	118 (98.3%)	215 (97.7%)	80 (96.4%)	
	Smoking history, but quitted	8 (1.9%)	1 (0.8%)	4 (1.8%)	3 (3.6%)	
Alcohol use	Non-drinker	211 (49.9%)	60 (50.0%)	112 (50.9%)	39 (47.0%)	0.85
	Drinker	212 (50.1%)	60 (50.0%)	108 (49.1%)	44 (53.0%)	
Time spend sitting in 1 day (hour) (Mean ± SD)		7.9 (4.8)	7.5 (4.6)	8.1 (5.0)	7.7 (4.6)	0.48
Mental health status	Fully recovered	15 (3.5%)	4 (3.3%)	9 (4.1%)	2 (2.4%)	0.37
	In treatment	14 (3.3%)	5 (4.2%)	4 (1.8%)	5 (6.0%)	
	Non-diagnosed	394 (93.1%)	111 (92.5%)	207 (94.1%)	76 (91.6%)	

^*^P-value < 0.05.

SD, standard deviation; PA, physical activity; U/D, underlying disease. Bold values indicate significant associations.

### 3.2 Physical activity and associated factors

The prevalence of recommended physical activity in high PM2.5 and low PM2.5 periods were 76.36 and 71.63%, respectively. While, poor physical activity in high PM2.5 and low PM2.5 periods were 23.64 and 28.37%, respectively. Table 2 shows the factors associated with recommend physical activity among the participants. Internal motivation for exercise was found to be significant in both low pollution (aOR = 2.46, 95% CI = 1.14 – 5.27; *p* = 0.021) and high pollution period (aOR = 4.00, 95% CI = 1.84 – 8.70; *p* < 0.001), with no significant difference observed between the estimated effects across these two periods. The another factor that was found to be the significant association only within the low pollution period was the outdoor exercise (aOR = 1.72, 95% CI = 1.03 – 2.87; *p* = 0.038). The remaining factors were found to be non-significant.

**Table 2:** Physical activity-associated factors among the participants during low and high PM2.5 period.

**Variable**		**Low PM2.5 periods**			**High PM2.5 periods**		
		**aOR**	**[95% CI]**	***p*** **-value**	**aOR**	**[95% CI]**	***p*** **-value**
Age (years)		1.14	0.98 – 1.32	0.081	1.03	0.90 – 1.18	0.641
Sex	Male	1 (base)					
	Female	0.90	0.55 – 1.48	0.676	0.64	0.37 – 1.08	0.094
BMI (kg/m^2^)		0.99	0.94 – 1.05	0.854	0.95	0.90 – 1.01	0.093
Underlying disease	Have U/D	1 (base)					
	No U/D	0.89	0.48 – 1.66	0.719	0.73	0.36 – 1.46	0.373
Smoking status	Non-smoking	1 (base)					
	Smoking	0.44	0.03 – 7.58	0.575	0.26	0.02 – 4.36	0.350
	Smoking history, but quitted	3.13	0.35 – 28.09	0.307	N/A	N/A	
Alcohol use	Non-drinker	1 (base)					
	Drinker	1.01	0.63 – 1.62	0.963	0.97	0.59 – 1.60	0.901
Mental health status	Non-diagnosed	1 (base)					
	Fully recovered	1.05	0.30 – 3.66	0.945	0.57	0.18 – 1.86	0.354
	In treatment	0.62	0.19 – 1.98	0.420	0.65	0.19 – 2.25	0.493
Sedentary lifestyle in 1 day (hour)		1.21	0.97 – 1.07	0.414	1.01	0.96 – 1.06	0.775
Faculty	Non-health sciences	1 (base)					
	Health sciences	1.21	0.69 – 2.11	0.513	0.99	0.53 – 1.85	0.984
Sufficient income	Sufficient	1 (base)					
	Insufficient	0.72	0.38 – 1.36	0.314	1.16	0.57 – 2.33	0.687
Internal motivation	No motivation	1 (base)					
	Have motivation	2.46	1.14 – 5.27	**0.021** ^*^	4.00	1.84 – 8.70	**< 0.001** ^**^
External motivation	No motivation	1 (base)					
	Have motivation	0.76	0.45 – 1.27	0.299	0.93	0.54 – 1.60	0.805
PM levels checked	Never checked	1 (base)					
	Always checked	1.44	0.56 – 3.70	0.455	0.71	0.36 – 1.39	0.317
	Often checked	1.50	0.93 – 2.42	0.099	0.91	0.52 – 1.59	0.744
Exercise location	Indoor exercise	1 (base)					
	Outdoor exercise	1.72	1.03 – 2.87	**0.038** ^*^	1.29	0.70 – 2.35	0.414

^*^P-value < 0.05. ^**^P-value < 0.001.

95% CI, 95% confidence interval; PA, physical activity; U/D, underlying disease; PM, particulate matter. Bold values indicate significant associations.

**Figure 2:**
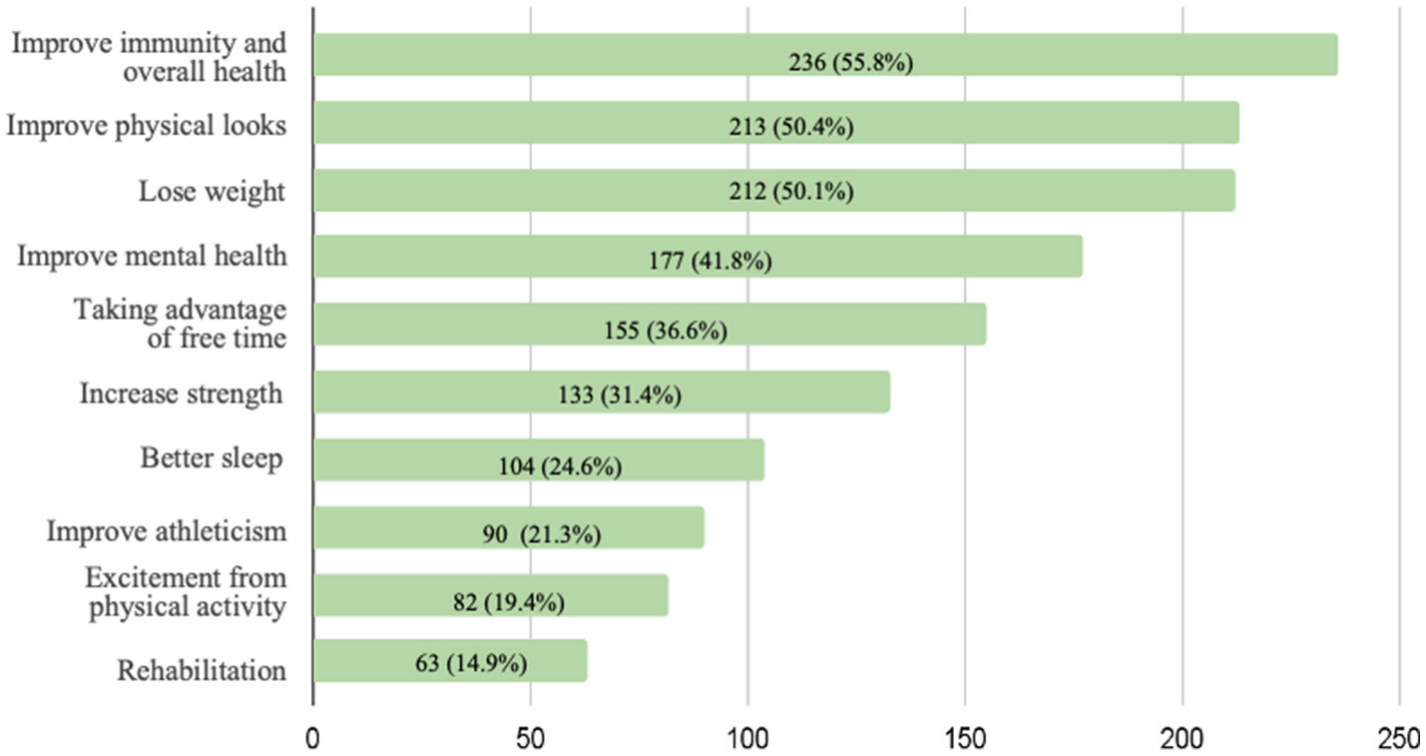
Frequency of internal motivation checked among the participants in low PM2.5 period.

As shown in Figure 2, the most reported internal motivation among participants was to improve immunity and overall health (55.8%) followed by improving physical skill (50.4%), losing weight (50.1%), and improving mental health (41.8%), respectively.

### 3.3 Physical activity between high and low PM2.5 period

**Figure 3:**
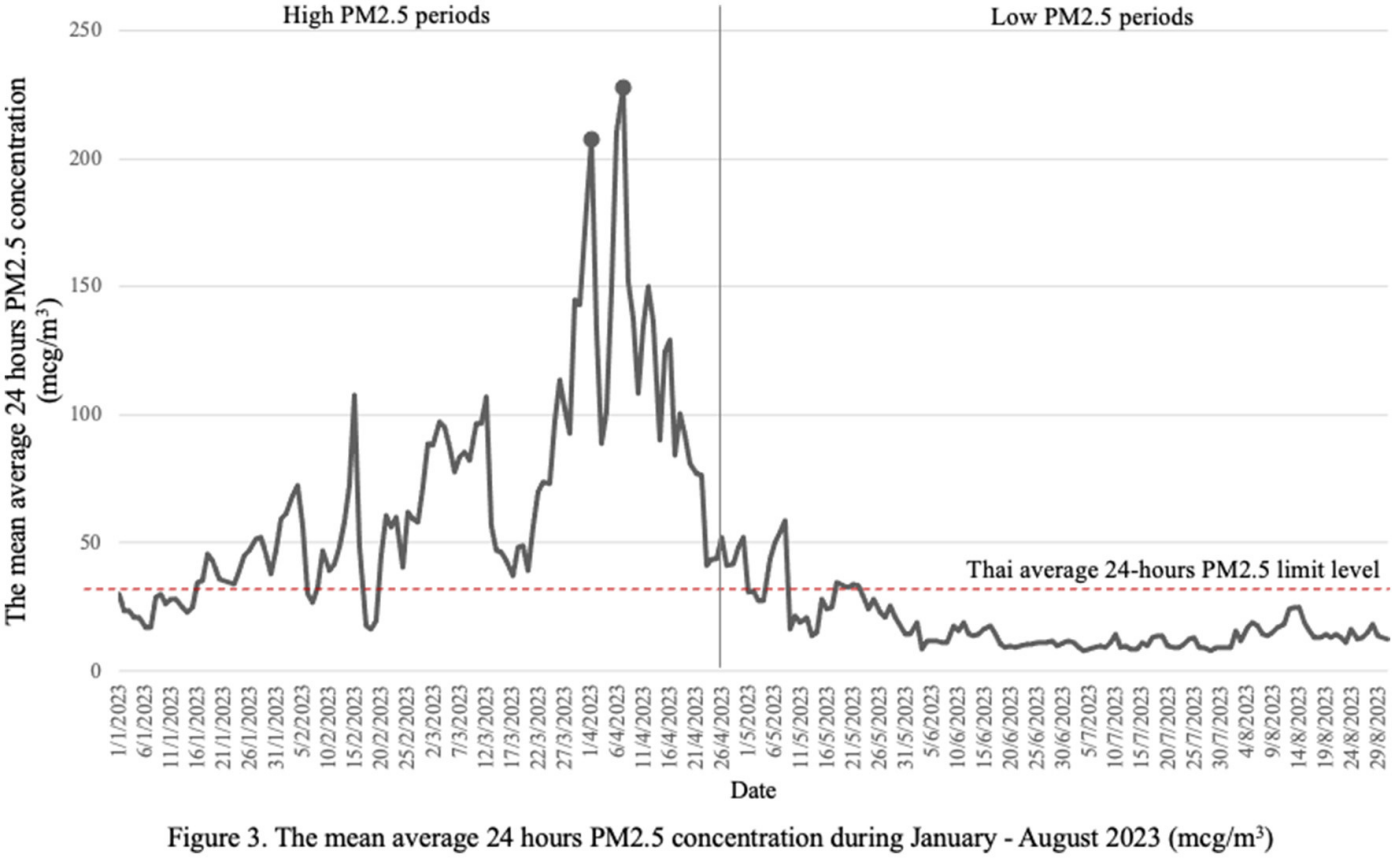
The mean average 24 h PM2.5 concentration during January—August 2023 (mcg/m^3^).

**Figure 4:**
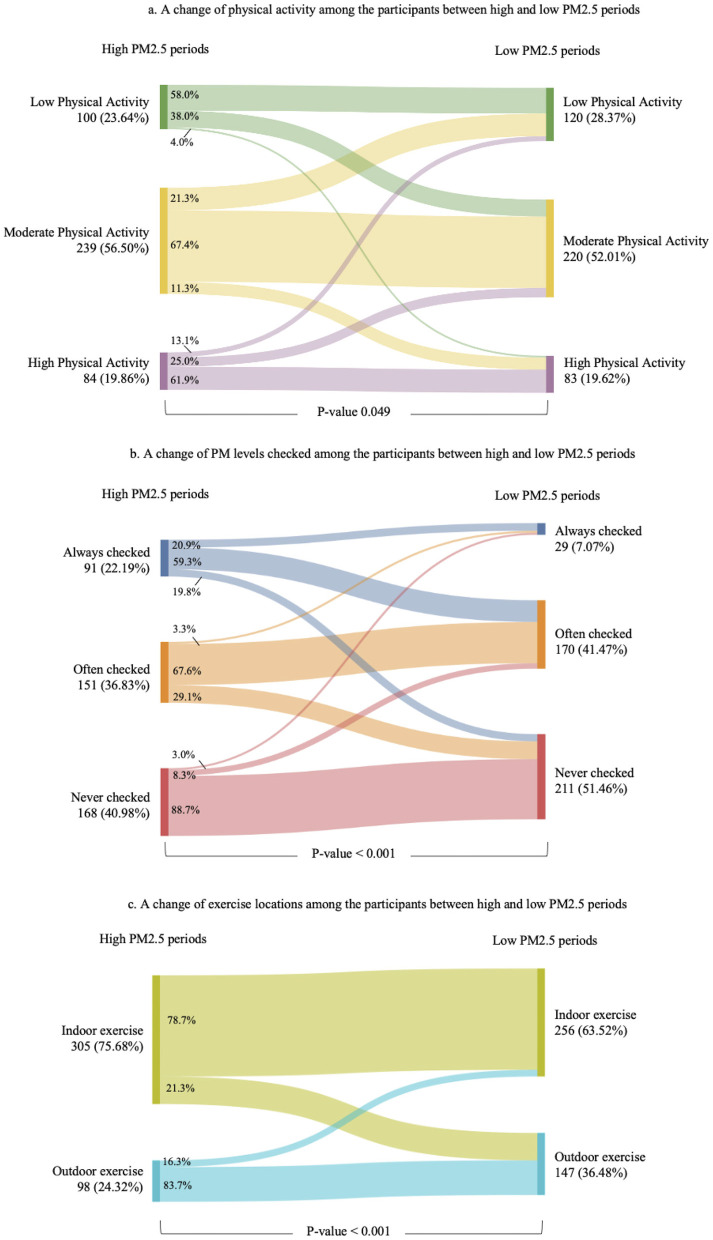
The Sankey diagrams showed a behavioral change for physical activity **(a)**, PM2.5 levels checked **(b)**, and exercise locations **(c)** among participant between high and low PM2.5 periods. The changes showed significant differences between the two periods for physical activity (*p* = 0.049), PM2.5 level checks (*p* < 0.001), and exercise locations (*p* < 0.001).

The mean average 24-hour PM2.5 concentration during high PM2.5 period and low PM2.5 period were 68.62 (43.03) and 16.79 (9.53) mcg/m^3^, respectively. The overall PM2.5 concentration is shown in Figure 3. The result showed positive and negative changes in the intensity of physical activity among the participants during high and low pollution periods with significant change *p*-value = 0.049 (Figure 4a). Figure 4b illustrates the participants' habits of checking PM2.5 levels during high and low PM2.5 periods. During high PM2.5 periods, a higher percentage of participants consistently checked PM2.5 levels, with 22.19% reporting always checking, compared to only 7.07% during low PM2.5 periods. This showed significant individual changes (*p* < 0.001). Figure 4c illustrates the distribution of locations for physical activity during both high and low PM2.5 periods. Notably, among the 305 participants who engaged in indoor exercise during the high PM2.5 period, 21.3% transitioned to outdoor exercise during the low PM2.5 period. There was a significant difference observed in the locations where physical activity occurred (*p* < 0.001).

## 4 Discussion

Our study provided an intuition into how PM2.5 level affected physical activity among university students in Chiang Mai. The current study was the first research conducted in Thailand targeting the Northern region which was seasonally pretentious by PM2.5 during the burning period (around February–May). The results may be beneficial in understanding clearer associations and variations in responding to air pollution in university student populations. We found that the prevalence of recommended physical activity decreased from 73.36% during the high PM2.5 period to 71.63% during the low PM2.5 period. This seemingly counterintuitive result compared with the findings in Beijing, China (9, 11, 25) and the meta-analysis which includes studies in the United Kingdom and the United States (20) where higher PM2.5 levels significantly reduced physical activity. Moreover, our study contrasts with previous studies that reported more sedentary behavior, such as increased sleep duration and reduced leisure-time physical activity, during high pollution periods (9, 26, 27). The reason for the different results could be discussed in terms of the type of physical activity that our study showed the shifting from outdoor to indoor physical activity during the pollution period due to air quality concerns. Furthermore, the period of the burning season matches with the summer break so the student may have more time to do physical activity than during the semester. The other reason that could relate to the lower physical activity was the rainy season which partially covered the non PM2.5 period. Supported by the study of Bélanger et al. that reported every 10 mm. of rainfall would decrease physical activity by 2–4% (28).

Our study demonstrated the significant association between the recommended physical activity and outdoor exercise in low pollution. The study by Calogiuri et al. (29) suggested that outdoor activity promoted the motivation to do more physical activity which supports our result. Interestingly, our other findings may demonstrate the new trends shifting to indoor physical activity during high PM2.5 periods underscore the adaptive behavior of students to reducing the health risk associated with poor air quality. Indoor facilities for doing physical activity such as fitness centers, gyms, or even fitness game consoles were getting more popular which may encourage students to spend more time exercising (30, 31). The outdoor activity may be also limited time by daylight which does not affect the indoor activity, for example, the fitness center tends to operate 24 h in Chiang Mai serving the new generation's lifestyle. The difference between indoor and outdoor physical activity should be an important issue to be extensively discussed in terms of physical, psychological, and social benefits (32).

The risks and benefits of physical activity and outdoor pollution were important issues to consider. The previous studies revealed the outweighing health benefits of physical activity over air pollution except for the extremely highly polluted environment (18, 19, 33). The study of Tainio et al. (19) conducted a sensitivity analysis, suggesting that during extremely high PM2.5 concentrations (100 μg/m3), the health risks may surpass the benefits after ~1 h and 30 min of cycling or 10 h of walking per day. While the beneficial effects of outdoor physical activity generally outweigh the harmful effects of air pollution, these benefits diminish when high-intensity physical activity is performed in a polluted environment due to its direct impact on the respiratory system and adverse effects on cardiovascular health (18, 33). Even the current study exhibited a high prevalence of recommended level of physical activity during high PM2.5 periods but most of the activities were indoors which may alleviate the health risk from air pollution. It is important for university and public policymakers to encourage indoor physical activity during high pollution periods. This can be achieved by providing indoor exercise facilities, offering discounts or free access to gyms, and implementing flexible benefit policies to promote the use of indoor facilities. These measures can help maintain physical activity levels while mitigating the adverse health effects of outdoor pollution.

The high prevalence of moderate to vigorous activity during both polluted and non-polluted periods among our study population may indicate good health literacy regarding the importance of physical activity. Shifting from outdoor to indoor activity is also evidence of good adaptive behavior in responding to the health risks from air pollution. This adaptation may occur in the situation that air pollution always happens at a certain time for many years in combination with individual resilience and adaptations leading to behavioral change. Internal motivation also might be the key to driving behavior change even in adverse conditions. Internal motivation, such as enjoyment and personal values, was a significant associated factor with physical activity among the participants according to the study about motivation for physical activity study in adolescents (34, 35). Exploring the internal motivation from our study found that, improving immunity and over health, improve physical looks and lose weight were the most reason to do physical activity. Understanding the motivation would facilitate in designing the health promotion programs among the same population setting elsewhere.

Additionally, the healthy worker bias should be considered in this study, as most participants were health science students. These students tend to have more health knowledge and may be healthier than the general population with different educational and socioeconomic backgrounds. Furthermore, participants who are more physically active may have been more inclined to join a study about physical activity, potentially leading to an overestimation of physical activity levels (36). The study found that students were more likely to check PM2.5 levels during high PM2.5 periods and might use them to decide where should they do physical activity. His heightened awareness indicates a proactive approach to managing exposure to air pollution, which is a crucial aspect of public health behavior. Developing tools for air quality monitoring and communication to warn people about air pollution-related health risks is essential (37, 38). For example, the study in Pittsburgh which provided portable indoor air quality monitors, along with a supportive web platform, empowers individuals to better understand and mitigate risks (38). These tools enable people to adopt healthier behaviors, enhancing their overall wellbeing. Thus, creating measures and warning policies in the organization such as stricter air quality regulations and promoting safe indoor exercise environments, can provide actionable insights.

The current air pollution crisis has intensified and spread more widely, primarily due to the impacts of climate change. This research underscores the urgent need for adaptation to unavoidable high PM2.5 levels. Relevant authorities must prioritize pollution control measures while simultaneously implementing health-promoting policies. By addressing pollution and promoting health concurrently, we can develop a comprehensive strategy that that supports physical activity during the air pollution period.

### 4.1 Strengths and limitation

This employed a validated Thai version of the IPAQ, ensuring reliable data collection. The analysis captures the high and low PM2.5 periods impacts on physical activity, offering valuable insights into behavior changes due to varying air quality. The findings highlighted adaptive behaviors, such as shifting to indoor activities during high pollution periods. Additionally, the study's focus on intrinsic motivation, such as the desire to improve health and immunity, provides a deeper understanding of the factors driving physical activity. However, there are notable limitations. The reliance on self-reported data introduces potential biases. The healthy worker effect might exist, the future study should be conducted with random or stratified sampling rather than the convenient sampling. More specific and standardized tools are needed for assessing physical activity motivation to better encourage improvement. The cross-sectional design limits the ability to determine causality, and the findings are specific to Chiang Mai University students, which may affect generalizability. The lack of longitudinal data restricts the examination of long-term trends and the sustained impact of air pollution on physical activity. The next study with detailed and accurate records of daily physical activity and air quality indices could significantly improve our understanding of the relationship between high PM2.5 levels and physical activity, providing a clearer and more detailed picture of this complex interaction.

## 5 Conclusion

This study was a marked preference for indoor exercise, a habit of consistently checking PM2.5 levels, and a higher prevalence of recommended physical activity during high PM2.5 periods. This adaptive behavior highlights the urgent need for policies promoting safe indoor exercise environments, effective pollution control measures, and air quality monitoring and warning tools. Internal motivation, such as the desire to improve health and immunity, played a significant role in maintaining physical activity levels. These findings underscore the importance of understanding and leveraging internal motivation in public health strategies to ensure continued physical activity even in adverse environmental conditions, ultimately enhancing public health resilience against environmental hazards.

## Data Availability

The data that support the findings of this study are available from the corresponding author upon reasonable request.
